# Exploring CALD and Non-CALD Women’s Behavioral and Dietary Responses to a Low-Intensity Intervention for Gestational Diabetes

**DOI:** 10.3390/nu17203191

**Published:** 2025-10-10

**Authors:** Kai Liu, Georgia S. Clarke, Melissa Oxlad, Jessica A. Grieger

**Affiliations:** 1 Adelaide Medical School, The University of Adelaide, Adelaide 5005, Australia; kai.liu@adelaide.edu.au (K.L.); georgia.clarke@adelaide.edu.au (G.S.C.); 2 Robinson Research Institute, The University of Adelaide, Adelaide 5005, Australia; 3 Lifelong Health Theme, South Australian Health and Medical Research Institute, Adelaide 5005, Australia; 4 School of Psychology, The University of Adelaide, Adelaide 5005, Australia; melissa.oxlad@adelaide.edu.au

**Keywords:** gestational diabetes, women, culturally and linguistically diverse, dietary advice, behavior change

## Abstract

Background/Objectives: Cultural backgrounds can shape dietary beliefs, food preferences, and attitudes toward health interventions. However, limited research has explored adherence or behavioral responses following a dietary intervention in women from culturally and linguistically diverse (CALD) backgrounds. This secondary analysis of a randomized controlled feasibility trial in women with gestational diabetes (GDM) explored differences in adherence, satisfaction, and behavior change between CALD and White (non-CALD) women. Methods: Thirty-eight participants were randomized to the dietary intervention (individualized, culturally tailored dietary advice) or standard care. Data collected at baseline (26–32 weeks’ gestation) and end of study (close to delivery) included a behavior change questionnaire, a 10-point Likert scale for adherence and satisfaction with the intervention, and 24-h dietary recall. Results: Eighteen participants self-reported as CALD and 20 self-reported as non-CALD. All intervention group participants, irrespective of cultural background, were motivated to make dietary changes, with similar mean [95% CI] adherence scores (CALD: 8.10 [7.27, 8.94] vs. non-CALD: 7.58 [6.66, 8.51]), and satisfaction scores to the intervention (CALD: 7.85 [6.96, 8.74] vs. non-CALD 6.88 [5.89, 7.86]). Within the intervention or standard care groups there were no differences in dietary intake between CALD and non-CALD participants. Conclusions: A low-intensity individualized dietary intervention for GDM was similarly acceptable and feasible for our small group of CALD and non-CALD participants. Findings support the potential for broadly applicable dietary strategies in antenatal care but also highlight the need for more culturally nuanced research to ensure inclusive interventions.

## 1. Introduction

Gestational diabetes mellitus (GDM) affects an increasing proportion of pregnant women globally [1]. Affected women have an increased risk for a range of short- and long-term adverse health outcomes, including preeclampsia, the delivery of babies large-for-gestational-age, and future risk for type 2 diabetes [2]. Dietary modification remains central to GDM management; however, the effectiveness of dietary interventions depends not only on the nutritional advice provided but also on the participant’s ability and willingness to engage with it [3].

Women from culturally and linguistically diverse (CALD) backgrounds have higher rates of GDM [4] and their infants are more likely to be born preterm, with low birthweight [5] or delivered via emergency Caesarean section [6]. In Australia, 1 in 3 pregnancies occur among women born outside of Australia, and the immigration rate is increasing [7,8]. Previous studies have highlighted barriers experienced by CALD women when accessing and engaging with antenatal care, including challenges around language, communication, and low food and health literacy [9,10]. Cultural background can also shape dietary beliefs, food preferences, and attitudes toward health interventions, potentially influencing both adherence to dietary advice and satisfaction with care [11,12]. Our previous qualitative analysis found that CALD women with GDM had low awareness of the condition and faced dietary challenges, often modifying traditional meals or preparing separate dishes to meet dietary recommendations for GDM [13]. However, there is limited research exploring how challenges experienced by CALD women affect behavior change and dietary intake following GDM diagnosis.

In our primary pilot study of 38 women with GDM, we tested the feasibility of a low-intensity dietary intervention designed to attenuate the rise in triglycerides [14]. The dietary advice was delivered in a personalized and culturally tailored manner to encourage nutrient-dense, minimally processed foods such as nuts, fruits and vegetables, and fish rich in omega-3 fatty acids, while reducing ultra-processed foods. The intervention was feasible and highly acceptable, with positive improvements in dietary behaviors and birthweight [15]. This secondary analysis aims to explore CALD and non-CALD women’s responses regarding motivation and perceived ability for behavior change, intervention adherence and satisfaction, and dietary intake. By enhancing our understanding of how cultural background influences dietary behaviors and intervention engagement, more effective, culturally responsive strategies could be developed to support women with GDM and to improve the equity and quality of antenatal care.

## 2. Materials and Methods

This study is a secondary analysis of a parallel-group, feasibility, randomized controlled trial in pregnant women diagnosed with GDM [15]. The study protocol was registered with the Australian and New Zealand Clinical Trial Registry on 24 February 2023 (ACTRN12623000199617p). Ethics approval was first obtained from the Women’s and Children’s Health Network Human Research Ethics Committee on 15 May 2023 (2023/HRE00008). The Consolidated Standards of Reporting Trials (CONSORT) reporting guidelines were followed and participant flow is reported in Figure S1.

### 2.1. Participant Recruitment and Randomization

We have published full study recruitment and eligibility details previously [15]. Briefly, participants were recruited from either gestational diabetes group education sessions at the Women’s and Children’s Hospital or through paid Facebook and Instagram advertisements on the University of Adelaide social media platforms. Inclusion criteria were English speaking, aged over 18 years, and current GDM diagnosis. Exclusion criteria were overt diabetes in pregnancy, type 1 or type 2 diabetes, poorly controlled hypothyroidism, Graves’ Disease, multiple pregnancy, vegetarian or vegan diet, and use of steroids, lipid-lowering drugs, antipsychotics, or antibiotics in the past 3 months. Permuted block randomization was performed to allocate eligible and consented participants to standard GDM care or the intervention group (i.e., standard GDM care + dietary intervention) in a 1:1 ratio using the Research Electronic Data Capture (REDCap) platform.

### 2.2. Study Protocol

All participants attended the research clinic at baseline (26–32 weeks’ gestation) and end of study (as close as possible to their estimated delivery date), either at the Women’s and Children’s Hospital or the South Australian Health and Medical Research Institute. Each clinic visit consisted of a behavior change questionnaire, a food questionnaire, a 5-step 24-h dietary recall, and collection of a non-fasting venous blood sample. Blood samples were collected using EDTA and fluoride oxalate vacuum tubes, centrifuged immediately at 4 °C, 1500 rpm, for 10 min, and frozen at −80 °C. For each participant, efforts were made to schedule blood tests at similar times of day at baseline and the end of the study. Demographic data (including self-reported cultural background, highest education level, and household income), and pregnancy-related data (including maternal age, pre-pregnancy body weight, gravidity, parity, consumption of alcohol and smoking in the 3 months before or during pregnancy, and supplement and medication use) were obtained using online questionnaires at the baseline visit. Pre-pregnancy body mass index (BMI) was calculated using height (measured by stadiometer at the research clinic) and self-reported pre-pregnancy body weight. Participants reported birth outcomes through a REDCap questionnaire with an uploaded photo of their infant birth page in the standard My Health and Development Record used in Australia two weeks following delivery.

### 2.3. Dietary Intervention

Standard care group: All participants received usual GDM management which consisted of dietary carbohydrate modification, self-blood glucose monitoring, and physical activity, with follow-up appointments when required [16].

Intervention group: In addition to usual GDM care, the intervention group received two individual dietary counselling sessions with the study dietitian (~30 min, scheduled as a face-to-face session or a telephone call for participant’s convenience). The study dietitian was trained and accredited in Australia and is of CALD background (Northeast Asian background). Dietary advice was provided based on our previous modelling study to alter triglycerides through dietary choices [14]. Care was taken to ensure dietary advice was culturally relevant and appropriate for each participant. A behavior change questionnaire was also administered for interim assessment of the intervention group. The first follow-up was a telehealth session in which dietary adherence was assessed, and the study dietitian provided lifestyle support using behavior change techniques such as goal setting, counterconditioning, reinforcement management and problem solving. Participants were contacted through text messages to obtain self-reported data on adherence to and satisfaction with the intervention using a 10-point Likert scale just before the first and second follow-up sessions (~30–33 weeks’ gestation and 32–35 weeks’ gestation, respectively), and at the end of the study (~37 weeks’ gestation). The intervention was considered low intensity, providing dietary advice similar to participant’s habitual diet, as well as limited participant contact time during the study period.

### 2.4. Study Outcomes

Adherence and satisfaction: Participant adherence to and satisfaction with the dietary intervention were assessed using separate 10-point Likert scales. For both scales, a score of 1 means lower adherence or satisfaction with the intervention and a score of 10 means higher adherence to or satisfaction with the intervention.

Behavior change: A behavior change questionnaire assessed perceived stages of dietary change, satisfaction and intention to change lifestyle habits, including dietary habits, physical activity, body weight, sleep, alcohol use and smoking. Participants’ perceived stages of dietary change were assessed by a single-answer, multiple-choice question adapted from the Transtheoretical Model of behavior change [17], which include stages of pre-contemplation—“know to improve diet but not intend to”, contemplation—“seriously intend to and will improve in the next 6 months”, preparation—“have plans to improve diet in the next month”, action—“currently improving diet” and maintenance—“changed diet and maintaining”. Participants were also asked to rate their perceived motivation and ability to change these behaviors using a 7-point Likert scale throughout the study period, where a score of 1 indicates lower perceived motivation or ability for behavior change, a score of 7 indicates greater perceived motivation or ability for behavior change, or “not applicable” if they considered these behaviors irrelevant to their pregnancy.

Dietary intake: From the 24-h recalls taken at baseline and end of study, total daily energy intake, protein, carbohydrates (including sugar, added sugar and dietary fiber), and fat (including saturated, mono- and poly-unsaturated fatty acids, linoleic acid, alpha-linoleic acid, and very long chain omega-3 fatty acids) were assessed using FoodWorks10 professional program (version 10.0.4266, Xyris Software).

### 2.5. Statistical Analysis

This secondary analysis adopted an available case approach whereby all available data for each variable were included in the analysis instead of a complete case analysis. Therefore, sample sizes varied across reported outcomes, with total numbers at each timepoint reported in the tables and figures. Participants were grouped by cultural background to explore potential differences in dietary adherence, satisfaction, and clinical outcomes in response to the intervention. Due to small sample sizes within individual cultural subgroups (e.g., Southeast Asian, Northeast Asian, Middle Eastern), the CALD participants were combined into a single group. This binary grouping (CALD vs. non-CALD) enabled meaningful comparison while preserving statistical power. The decision to aggregate was based on the exploratory nature of the analysis and the feasibility design of the original study, rather than an assumption of cultural homogeneity.

Descriptive data are reported as mean [standard deviation, SD] or percentages for continuous or categorical variables, respectively. Visual inspection from Q-Q plots and histograms were used to check the VAS data for normality. Dietary intake at the end of the intervention was compared across cultural group and study group using a two-way ANOVA, adjusting for the corresponding baseline dietary intake. Model-derived estimated marginal means alongside corresponding 95% confidence intervals are reported. A linear mixed-effects model was used to test whether dietary adherence to or satisfaction with the dietary intervention, and the perceived motivation and ability for behavior change differed between ethnic groups. Data were analyzed using SPSS (version 29, IBM Corp., New York, NY, USA).

## 3. Results

Thirty-eight participants completed the study. Twenty participants (52.6%) self-reported to be White (Australian, European), and 18 self-reported to be of CALD background, including 13 (34.2%) with Asian background (*n* = 5 Northeast Asian, *n* = 3 Southeast Asian, *n* = 5 Southern or Central Asian), two (5.3%) reported to be Indigenous (Aboriginal or Torres Strait Islander), two (5.3%) reported to be American (Central or Sothern American) and one woman (2.6%) reported to be of African background. Demographic characteristics across all self-reported ethnicities are reported in Table S1.

For the broader CALD and non-CALD groups, the mean [SD] age was 33.3 [4.56] years for CALD women and 31.4 [4.74] years for non-CALD women. The mean [SD] BMI was 25.6 [6.34] kg/m^2^ among CALD women and 33.1 [8.30] kg/m^2^ among non-CALD women. Baseline characteristics of CALD and non-CALD participants randomized to the standard care and intervention groups are presented in Table 1.

### 3.1. Perceived Motivation and Ability for Behavior Change

**Figure 1:**
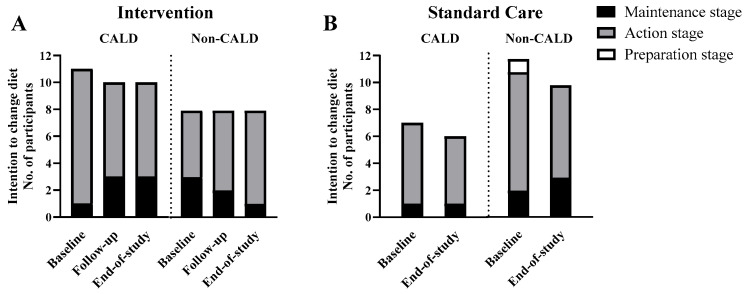
Number of CALD and non-CALD participants in the intervention (**A**) and standard care (**B**) groups at different stages of dietary change over the study.

**Figure 2:**
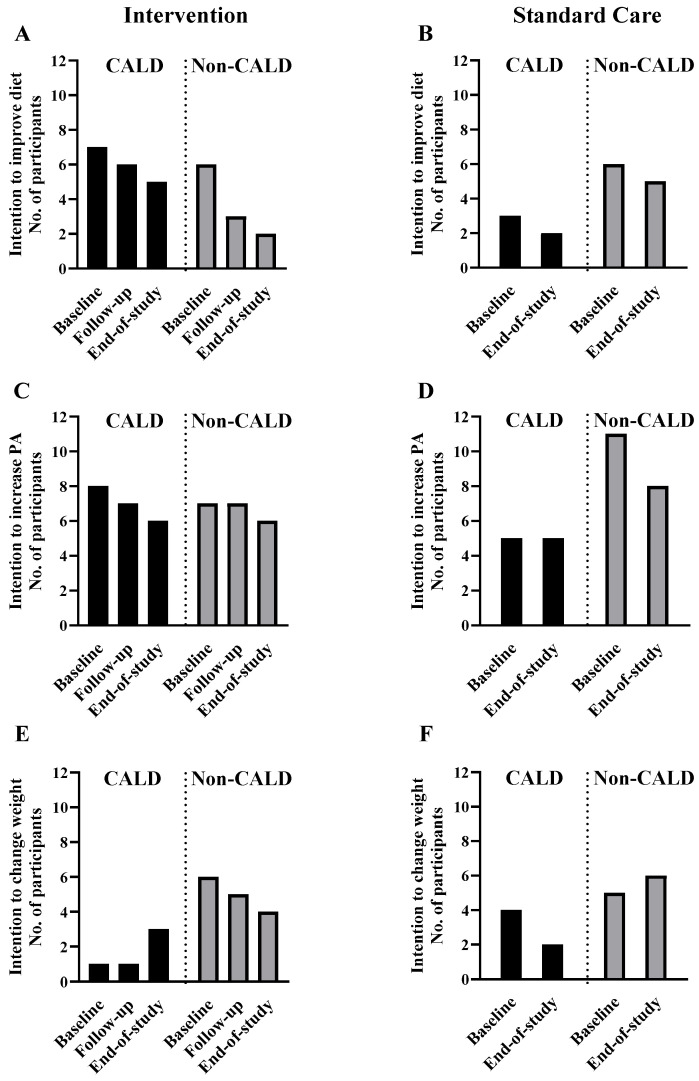
Number of CALD and non-CALD participants in the intervention and standard care groups prioritizing dietary habits (**A**,**B**), physical activity (**C**,**D**) and body weight (**E**,**F**) over the study.

**Figure 3:**
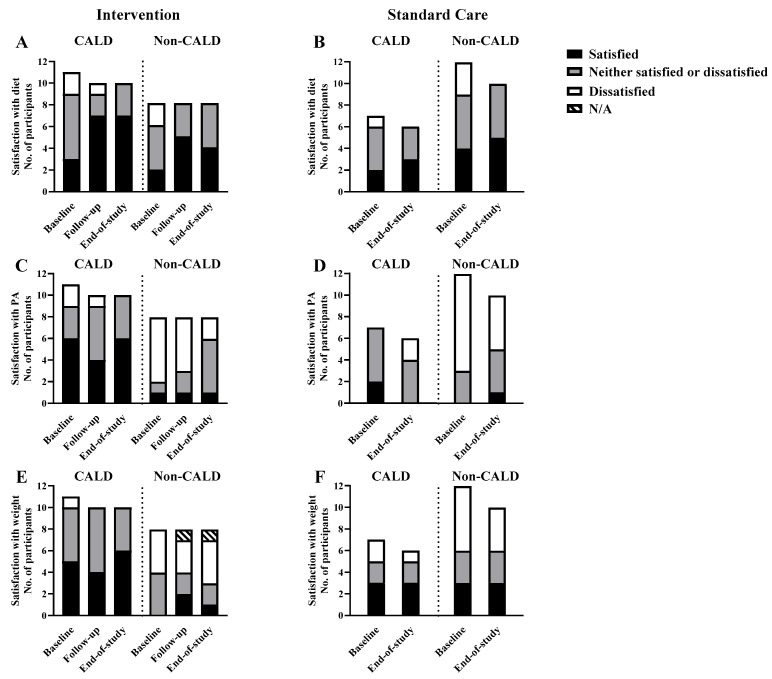
Number of CALD and non-CALD participants in the intervention and standard care groups reporting on satisfaction with their dietary habits (**A**,**B**), physical activity (**C**,**D**) and body weight (**E**,**F**) over the study.

The number of participants reporting the different stages of dietary change over the intervention is shown in Figure 1. No participants were in the pre-contemplation or contemplation phase. Nearly all participants (37 out of 38 (97%) at baseline and all 34 (100%) at the end of the study) reported to be in the action or maintenance stage to change their diet. In the intervention group, a higher number of CALD participants maintained the intention to improve their diet over the study (7 out of 11 (64%) at baseline to 5 out of 10 (50%) at the end of the study) compared to the larger decrease in the number of non-CALD participants intending to maintain their diet at the end of the study (6 out of 8 (75%) at baseline to 2 out of 8 (25%) at the end of study) (Figure 2A). Likewise, dietary satisfaction showed a higher number of participants in the intervention group, particularly for CALD participants (3 out of 11 (27%) at baseline to 7 out of 10 (70%) at the end of the study) than non-CALD participants (2 out of 8 (25%) at baseline to 4 out of 8 (50%) at the end of the study (Figure 3A). In the standard care group, satisfaction and priority for dietary change decreased for CALD and non-CALD participants (Figure 3B).

Although physical activity was not a major focus in this pilot study, a higher number of CALD participants reported satisfaction with their physical activity in the intervention group (6 out of 11 (55%) at both baseline and end of study), a pattern less evident in non-CALD participants (1 out of 8 (13%) at both baseline and end of study). More non-CALD participants were dissatisfied with their physical activity in both intervention and standard care groups (Figure 3C,D). Across both study groups, a higher number of non-CALD than CALD participants were dissatisfied with their body weight and intended to change it (Figure 3E,F). No clear patterns were observed over time for satisfaction or intentions regarding sleep, time spent with family/friends, alcohol or smoking (Table S2).

Linear mixed models showed no significant cultural group by time effect in perceived motivation (intervention: *p* = 0.59; standard care: *p* = 0.25) or ability to change dietary habits (intervention: *p* = 0.12; standard care: *p* = 0.80). However, within the intervention group, there was a slightly reduced perceived ability to change dietary habits in non-CALD participants overtime (median score 6 at baseline to 4 at end of the study) (Table S3), whereas a modestly increased perceived ability to change dietary habits in CALD participants (median score 4 at baseline to 6 at end of the study. For the standard care group, there was no change in perceived motivation or ability (Table S3) to change dietary habits among CALD and non-CALD participants. No clear patterns were found in perceived motivation and ability to change physical activity, body weight, sleep, time spent with family/friends, alcohol use or smoking over time across the different ethnic groups (Table S3).

### 3.2. Self-Reported Adherence to and Satisfaction with the Intervention

Self-reported adherence to and satisfaction with the dietary intervention is reported in Supplementary Table S4. There was no significant main effect of cultural background on adherence scores (F [1.0, 16.1] = 0.78, *p* = 0.39), and no significant interaction between cultural background and timepoint (F [4.0, 15.0] = 1.13, *p* = 0.38), indicating that adherence levels did not differ significantly between cultural groups or change differentially over time. Estimated marginal means [95% CI] suggest slightly higher adherence in the CALD (8.10 [7.27, 8.94] vs. non-CALD group (7.58 [6.66, 8.51]), but this was not statistically significant. Across timepoints, adherence remained relatively stable, with both groups reporting consistently higher mean scores (range: 7.25–8.35) (Supplementary Table S4).

Likewise, there was no significant main effect of ethnicity on self-reported satisfaction scores (F [1.0,16.3] = 2.43, *p* = 0.138), nor a significant interaction between ethnic groups and timepoint (*p* = 0.40). Estimated marginal means (95% CI) indicate that CALD participants reported slightly higher satisfaction (7.85 [6.96, 8.74]) compared to non-CALD participants (6.88 [5.89, 7.86]), but this was not statistically significant. Mean satisfaction in both groups was moderately high and remained relatively stable across time.

### 3.3. Dietary Intakes in CALD Vs. Non-CALD Participants

There was no difference in baseline dietary intakes between CALD and non-CALD participants (Table S5). In both the crude analysis and after adjusting for baseline intakes, there were no significant main effects of study group or cultural group on energy intake and most other nutrients at study completion, nor a significant interaction effect.

End of study energy and macronutrient intakes for the intervention and standard care groups and for the cultural groups are presented in Table S6. Compared with the standard care group, CALD participants in the intervention group had a lower sugar intake at the end of the study (mean [95% CI] 95.6 [72.8, 118.4] g vs. 53.6 [35.1, 72.2] g, *p* = 0.01) and the significance remained after adjusting for baseline sugar intake (93.7 [69.4, 118.0] g vs. 54.8 [35.4, 74.1] g, *p* = 0.019). CALD participants in the intervention group had a higher end of study monounsaturated fatty acids intake compared to the standard care group (50.2 [41.4, 58.9] g vs. 32.3 [17.0, 47.3] g, *p* = 0.04]), but the significance was lost after adjusting for baseline monounsaturated fatty acids intakes.

## 4. Discussion

This secondary analysis explored CALD and non-CALD participants’ responses to a low-intensity dietary intervention for GDM, assessing adherence, satisfaction, perceived behavior change, and dietary intake. No significant differences were found between groups; however, some important findings warrant discussion.

Self-reported adherence to and satisfaction with the dietary intervention did not differ significantly by cultural group, indicating that the intervention was broadly acceptable for CALD and non-CALD participants. This finding was somewhat unexpected given the unmeasured but potential differences in health literacy and food preferences among CALD participants [9,10]. Although satisfaction scores were marginally higher for CALD participants, this trend was not statistically significant. Broad acceptability was likely due to the intervention being low-intensity, personalized, and culturally considerate. These findings are encouraging given that previous research has reported the frequent absence of culturally tailored dietary advice in GDM nutrition care [13,18,19]. However, despite participants’ positive attitudes towards the dietary intervention, there were limited differences in energy and macronutrient intake between study groups among both CALD and non-CALD participants. The short duration and low intensity of the dietary intervention may have limited the effect on overall nutritional intake. Furthermore, although participants, including CALD participants, are motivated to change and improve their nutritional intake during pregnancy, anxiety and mental health challenges, along with social and cultural challenges associated with GDM management [11,13] highlight the ongoing need for culturally sensitive dietary advice and culturally competent care. The current findings suggest that a more flexible and individualized approach may mitigate some of these barriers while preserving intervention impact.

The perceived stages of dietary change indicated that most CALD and non-CALD participants were either in the action phase or maintenance phase of changing their diet, as described by the Transtheoretical model [17]. These two phases indicate advanced levels of engagement with behavior change, which may reflect the participants’ determination to improve their health. Although the current study did not explore the facilitators behind the dietary modifications, previous research has reported that participants across different cultural groups often have a strong intention to minimize the potential adverse pregnancy and birth outcomes associated with GDM [11,20,21]. A GDM diagnosis may therefore be a powerful motivator for all cultural groups to improve their diet and engage in education that is culturally relevant.

In the primary trial, lifestyle modification was a central focus emphasized by both health professionals and the research team. The culturally tailored dietary advice, which is highly valued but often lacking in routine GDM education in Australia [11,13], may have enhanced CALD participants’ motivation and perceived ability to change their diet. Comparatively, non-CALD participants prioritized physical activity and body weight management (as well as diet), possibly related to their higher BMI. The current study did not explore the potential reasons behind the discrepancy in priorities between CALD and non-CALD women. However, the greater focus on weight management and higher BMI in non-CALD women may reflect a potential internalized weight stigma, an adoption of self-directed negative weight stereotypes which is prevalent in “Western” populations [22]. These differences highlight the importance of incorporating diverse cultural needs and priorities into GDM care models to ensure effective, culturally sensitive support.

A key strength of this study was the inclusion of CALD participants, a group often underrepresented in nutrition and pregnancy interventions. The inclusion of both behavioral and dietary measures enabled a comprehensive assessment of intervention engagement beyond self-reported satisfaction. Another strength was the delivery of dietary counseling by a dietitian of CALD background which may have enhanced rapport and cultural sensitivity in communication, contributing to the high acceptability observed among CALD participants.

A key limitation of this analysis was the grouping of culturally diverse participants into a single CALD category. While this approach was necessary due to the small numbers in individual cultural subgroups, it likely masked differences in dietary intakes, intervention engagement, and cultural attitudes toward food that may exist across specific groups. As such, findings should be interpreted with caution and viewed as exploratory. As a secondary analysis, the study was underpowered to detect modest differences between groups.

## 5. Conclusions

This secondary analysis suggests that a low-intensity, dietary intervention may be equally acceptable and feasible for this small group of CALD and non-CALD women. Future studies with larger and more culturally stratified samples are needed to better understand how specific cultural contexts influence dietary behavior and responses to interventions among women with GDM.

## Figures and Tables

**Table 1:** Baseline characteristics among CALD and non-CALD participants randomized to the standard care or intervention group.

Variables	Standard Care		Intervention	
	CALD (*n* = 7)	Non-CALD (*n* = 12)	CALD (*n* = 11)	Non-CALD (*n* = 8)
Mean age, years (mean ± SD)	30.4 ± 4.8	31.6 ± 5.4	35.2 ± 3.5	31.1 ± 4.2
Pre-pregnancy BMI, kg/m^2^ (mean ± SD)	30.2 ± 7.5	33.4 ± 7.9	22.7 ± 3.2	32.7 ± 9.4
Highest education level, *n* (%)				
Completed secondary education, Yes ^1^	1 (14.3)	4 (33.3)	1 (9.1)	1 (12.5)
Completed tertiary education (undergraduate or postgraduate degrees), Yes (%)	5 (42.9)	8 (66.7)	10 (81.8)	7 (87.5)
Employed during pregnancy, n (%)	7 (100)	6 (50)	10 (90.9)	7 (87.5)
Gravidity, *n* (%)				
Primigravida	3 (42.9)	3 (25.0)	3 (27.3)	2 (25.0)
Multigravida	4 (57.1)	9 (75.0)	8 (72.7)	6 (75.0)
Parity, *n* (%)				
Nulliparous	3 (42.9)	5 (41.7)	4 (36.4)	5 (62.5)
Primiparous	3 (42.9)	6 (50.0)	5 (45.5)	2 (25.0)
Multiparous	1 (14.3)	1 (8.3)	2 (18.2)	1 (12.5)
Annual household income ($AUD), *n* (%)				
<$40,000	1 (14.3)	3 (25.0)	2 (18.2)	0 (0)
$40,001–$70,000	2 (28.6)	1 (8.3)	1 (9.1)	2 (25.0)
$70,001–$105,000	2 (28.6)	4 (33.3)	2 (18.2)	3 (37.5)
$≥105,001–$205,000	2 (28.6)	4 (33.3)	4 36.4)	3 (37.5)
Prefer not to disclose	0 (0)	0 (0)	2 (18.2)	0 (0)
Alcohol consumption, *n* (%)				
3 months leading up to pregnancy, Yes	3 (42.9)	9 (25.0)	5 (45.5)	6 (75.0)
During pregnancy, No	7(100)	12 (100)	11 (100)	8 (100)
Smoking, *n* (%)				
3 months leading up to pregnancy, Yes	1 (14.3)	0 (0)	0 (0)	0 (0)
During pregnancy, No	7 (100)	12 (100)	11 (100)	8 (100)

^1^ One CALD participant did not complete secondary education.

## Data Availability

Data are available on request from the authors.

## Supplementary Materials

The following supporting information can be downloaded at: https://www.mdpi.com/article/10.3390/nu17203191/s1, Figure S1: Study timeline; Figure S2: Flow diagram of participants in the study; Table S1: Baseline characteristics of study participants from each ethnicity group; Table S2: Satisfaction and intention to change lifestyle habits in CALD and non-CALD participants randomized to intervention and standard care groups; Table S3: Median Likert score of the perceived motivation and ability for behavior change in CALD and non-CALD participants; Table S4: Self-reported adherence to and satisfaction with the dietary intervention, using a 10-point Likert Scale, among CALD and non-CALD participants in the intervention group; Table S5: Baseline macronutrient intake in CALD and non-CALD participants; Table S6: Adjusted mean (95% CI) nutrient intakes at end of study, between CALD and non-CALD participants randomized to the intervention or standard care group.

## Abbreviations

The following abbreviations are used in this manuscript:

CALD	Culturally and linguistically diverse
CI	Confidence Interval
GDM	Gestational diabetes
