# Supplementary material for: Exploring CALD and Non-CALD Women’s Behavioral and Dietary Responses to a Low-Intensity Intervention for Gestational Diabetes
Source: Nutrients. 2025 Oct 10;17(20):3191. doi: 10.3390/nu17203191 (PMC12567258; doi:10.3390/nu17203191)
Supplement: Supplementary file 1 [file nutrients-17-03191-s001.zip › nutrients-3886450-supplementary.pdf]

Table S1. Baseline characteristics of study participants from each ethnicity group

	White (*n*=20)	Indigenous (*n*=2)	Northeast Asian (*n*=5)	Southeast Asian (*n*=3)	Southern and Central Asian (*n*=5)	Other (*n*=3)[a]
**Age, years, mean (SD)**	31.4 (4.74)	30.0 (4.24)	35.0 (3.08)	32.3 (2.08)	36.0 (3.74)	29.3 (7.51)
**Pre-pregnancy BMI, kg/m^2, mean (SD)**	33.1 (8.30)	38.9 (7.42)	25.7 (4.22)	20.2 (3.10)	22.9 (1.60)	26.7 (4.65)
**Highest education level, n (%)**						
Completed secondary education, Yes[b]	5 (25)	1 (100)	0 (0)	1 (33)	0 (100)	0 (0)
Completed tertiary education (undergraduate or postgraduate degrees), Yes	15 (75)	0 (0)	5 (100)	2 (67)	5 (100)	3 (100)
**Employed during pregnancy, *n* (%)**	13 (65)	2 (100)	5 (100)	3 (100)	4 (80)	3 (100)
**Gravidity, n (%)**						
Primigravida	5 (25)	0 (0)	1 (20)	2 (67)	1 (20)	2 (67)
Multigravida	15 (75)	2 (100)	4 (80)	1 (33)	4 (80)	1 (33)
**Parity, n (%)**						
Nulliparous	10 (50)	0 (0)	1 (20)	2 (67)	2 (40)	2 (67)
Primiparous	8 (40)	1 (50)	4 (80)	0 (0)	2 (40)	1 (33)
Multiparous	2 (10)	1 (50)	0 (0)	1 (33)	1 (20)	0 (0)
**Annual household income ($AUD), *n* (%)**						
<$40,000	3 (15)	0 (0)	1 (20)	0 (0)	2 (40)	0 (0)
$40,001 - $70,000	3 (15)	0 (0)	1 (20)	0 (0)	1 (20)	1 (33)
$70,001 - $105,000	7 (35)	1 (50)	1 (20)	1 (33)	0 (0)	1 (33)
$105,001 - $205,000	7 (35)	1 (50)	2 (40)	2 (67)	1 (20)	0 (0)
>$205,000	0 (0)	0 (0)	0 (0)	0 (0)	0 (0)	0 (0)
Prefer not to disclose	0 (0)	0 (0)	0 (0)	0 (0)	1 (20)	1 (33)
**Alcohol consumption, *n* (%)**						

3 months leading up to pregnancy, Yes	15 (75)	0 (0)	4 (80)	1 (33.3)	3 (60)	2 (66.6)
During pregnancy, No	20 (100)	2 (100)	5 (100)	3 (100)	5 (100)	3 (100)
**Smoking, *n* (%)**						
3 months leading up to pregnancy, Yes						
During pregnancy, No	0 (100)	0 (100)	0 (100)	0 (100)	0 (100)	0 (100)

[a] "Other" includes self-report of South American, Central American, or South African; [b] One Indigenous participant did not complete secondary education

Table S2. Satisfaction and intention to change lifestyle habits in CALD and non-CALD participants randomized to intervention and standard care groups

	Intervention						Standard Care			
	Baseline		Follow-up		End of study		Baseline		End of study	
	CALD (*n*=11)	non-CALD (*n*=8)	CALD (*n*=10)	non-CALD (*n*=8)	CALD (*n*=10)	non-CALD (*n*=8)	CALD (*n*=7)	non-CALD (*n*=12)	CALD (*n*=6)	non-CALD (*n*=10)
**Satisfaction with sleep, *n*:**										
Yes	6	5	5	4	5	4	1	4	1	2
Neither satisfied nor dissatisfied	3	1	2	4	3	2	5	3	3	4
No	2	2	1	2	2	2	1	5	2	4
N/A	0	0	0	0	0	0	0	0	0	0
**Satisfaction with time with family/friends, *n*:**										
Yes	7	5	6	6	7	6	4	5	4	7
Neither satisfied nor dissatisfied	4	2	0	2	2	1	3	5	2	1
No	0	1	2	1	1	1	0	2	0	1
N/A	0	0	0	1	0	0	0	0	0	1
**Satisfaction with alcohol consumption, *n*:**										
Yes	6	5	1	4	4	2	3	8	2	4
Neither satisfied nor dissatisfied	0	2	1	0	0	0	0	1	0	1
No	0	1	0	0	0	0	2	0	0	1
N/A	5	0	6	6	6	0	2	3	4	4
**Satisfaction with smoking, *n*:**										
Yes	5	5	2	3	4	2	3	8	2	3
Neither satisfied nor dissatisfied	0	0	0	0	0	0	0	0	0	0
No	0	0	0	0	0	0	1	0	0	1
N/A	6	3	6	7	6	6	3	4	4	6
**Intention to change lifestyle habits, *n*:**										
Sleep habits	10	4	2	5	5	3	4	7	3	6
Time with family/friends	5	3	3	4	4	3	2	5	1	2
Alcohol drinking	0	0	0	0	0	0	1	0	0	0
Smoking	0	0	0	0	0	0	0	0	0	0

Table S3. Median Likert score of the perceived motivation and ability for behaviour change in CALD and non-CALD participants

	Intervention						Standard Care			
	Baseline		Follow-up		End-of-study		Baseline		End-of-study	
	CALD (*n*=11)	non-CALD (*n*=8)	CALD (*n*=10)	non-CALD (*n*=8)	CALD (*n*=10)	non-CALD (*n*=8)	CALD (*n*=7)	non-CALD (*n*=12)	CALD (*n*=6)	non-CALD (*n*=10)
**Motivation to:**										
Change sleep	4	3.5	6	4	6	6	5	6	5	5
Change time with family/friends	4	3	6	4	5.5	4.5	5	4	4	5
Change dietary habits	6	6	6	5	6	5	5	5.5	4	5
Change physical activity	5	5	5	4.5	5.5	5	5	5.5	5.5	5
Change alcohol consumption	1	1	1	1	3.5	1	2.5	1	1	1
Change smoking status	1	1	1	1	3.5	1	1	1	1	1
Change weight	4.5	4.5	4	4	5.5	4.5	4.5	5	4.5	5
**Ability to:**										
Change sleep habits	5.5	4	5	5	5	2.5	4	5	2.5	4.5
Change time with family/friends	4	5	5	5	5	4	4	5	4.5	5.5
Change dietary habits	4	6	6	5	6	4	6	6.5	5.5	6
Change physical activity	5	5	5.5	5	4.5	3.5	5	5	4.5	5
Change alcohol consumption	7	6.5	4	7	5	1	2.5	1	7	4
Change smoking status	7	7	4	4	5	1	1	1	7	4
Change weight	5	3	4	5	4	4	4.5	5	3.5	5

Table S4. Self-reported adherence to and satisfaction with the dietary intervention, using a 10-point Likert Scale, among CALD and non-CALD participants in the intervention group

	**Intervention**	
	**CALD (*n*=10)**	**non-CALD (*n*=8)**
**Adherence**		
30-33 weeks' gestation	7.60 (1.43)	7.88 (2.10)
32-35 weeks' gestation	8.19 (1.0)	7.25 (1.67)
37 weeks' gestation	8.35 (1.16)	7.63 (1.41)
**Satisfaction**		
30-33 weeks' gestation	7.40 (1.58)	6.88 (2.42)
32-35 weeks' gestation	7.75 (1.28)	7.25 (1.75)
37 weeks' gestation	8.30 (1.57)	6.50 (1.51)

Data presented as mean (standard deviation). A higher score out of 10 indicates higher adherence to or satisfaction with the intervention.

Table S5. Baseline macronutrient intake in CALD and non-CALD participants

	CALD (*n*=18)	non-CALD (*n*=20)
Energy (kJ)	8988 (7517, 10459)	8740 (7649, 9830)
Protein (g)	114.7 (93.8, 135.9)	107.0 (92.3, 121.8)
Total fat (g)	90.9 (69.9, 111.8)	99.6 (81.4, 117.7)
Saturated fat (g)	30.5 (20.5, 40.5)	34.6 (27.5, 41.7)
PUFA (g)	14.6 (10.5, 18.7)	15.7 (10.6, 20.8)
MUFA (g)	42.1 (30.8, 53.5)	38.2 (26.0, 50.5)
Carbohydrate (g)	203.4 (165.4, 241.5)	180.0 (158.4, 201.6)
Sugars (g)	73.2 (59.9, 86.5)	74.6 (60.8, 88.3)
Added sugars (g)	24.6 (5.68, 43.6)	15.8 (7.61, 23.9)
Dietary fibre (g)	27.8 (22.7, 32.0)	30.8 (23.6, 38.0)
% protein	21.8 (19.3, 24.4)	20.3 (18.5, 22.2)
% total fat	36.8 (33.1, 40.5)	41.1 (36.6, 45.6)
% saturated fat	11.9 (9.68, 14.1)	14.3 (12.4, 16.3)
% carbohydrate	37.9 (33.2, 42.6)	34.9 (30.3, 39.5)
% fibre	2.56 (2.21, 2.92)	2.81 (2.20, 3.42)
Linoleic acid (g)	12.60 (8.37, 16.8)	13.2 (9.12, 17.4)
ALA (g)	1.68 (1.07,2.30)	1.94 (1.14, 2.74)
Very long chain omega-3 fatty acids (mg)	268 (48.8, 488)	524 (-207, 1255)
EPA (mg)	73.7 (-2.85, 150)	163 (-96.1, 422)
DPA (mg)	85.3 (34.8,136)	109 (-12.4, 230)
DHA (mg)	109 (-5.75, 224)	252 (-102, 607)

Data presented as mean (95% CI). **PUFA** polyunsaturated fatty acids; **MUFA** monounsaturated fatty acids; **ALA** α-linolenic acid; **EPA** eicosapentaenoic acid; **DPA** docosapentaenoic acid; **DHA** docosahexaenoic acid.

Table S6. Adjusted mean (95% CI) nutrient intakes at end of study, between CALD and non-CALD participants randomized to the intervention or standard care group

	CALD		Non-CALD	
	**Intervention (*n*=6)**	**Standard Care (*n*=9)**	**Intervention (*n*=8)**	**Standard Care (*n*=10)**
**Energy (kJ)**	8808 (8056, 9561)	8957 (8042, 9872)	8293 (7500, 9086)	8610 (7901, 9320)
**Protein (g)**	103.0 (81.9, 124.0)	116.2 (91.0, 141.3)	106.9 (85.0, 128.8)	98.0 (78.3, 117.6)
**Total fat (g)**	105.8 (91.1, 120.4)	91.4 (73.4, 109.3)	93.0 (77.4, 108.5)	92.4 (78.5, 106.3)
**Saturated fat (g)**	29.9 (23.0, 36.7)	36.6 (27.5, 45.8)	30.9 (23.8, 38.1)	36.0 (29.6, 42.3)
**PUFA (g)**	16.4 (11.7, 21.0)	13.0 (5.59, 20.4)	14.5 (8.30, 20.7)	12.0 (7.36, 16.6)
**MUFA (g)**	50.5 (39.4, 61.6)	33.5 (16.8, 50.2)	41.3 (27.0, 55.5)	41.5 (30.7, 52.4)
**Carbohydrate (g)**	172.3 (142.1, 202.5)	197.7 (161.3, 234.1)	170.4 (138.4, 202.5)	190.9 (162.7, 219.1)
**Sugars (g)**	**54.8 (35.4, 74.1)***	**93.7 (69.4, 118.0)**	76.6 (56.4, 96.8)	86.0 (68.1, 104.0)
**Added sugars (g)**	14.1 (-2.48, 30.7)	35.8 (13.4, 58.2)	24.9 (6.35, 43.4)	20.7 (6.62, 34.8)
**Dietary fibre (g)**	29.2 (21.3, 37.0)	27.3 (17.5, 37.1)	28.9 (20.0, 37.8)	29.1 (21.6, 36.7)
**% protein**	19.6 (16.3, 22.9)	21.6 (17.6, 25.6)	22.1 (18.6, 25.5)	19.4 (16.1, 22.7)
**% total fat**	43.4 (38.6, 48.2)	37.4 (31.6, 43.2)	42.1 (36.9, 47.2)	41.6 (36.8, 46.3)
**% saturated fat**	12.1 (9.0, 15.2)	15.4 (11.4, 19.3)	14.3 (11.1, 17.5)	16.0 (13.1, 18.9)
**% carbohydrate**	33.5 (28.4, 38.6)	36.9 (30.7, 43.2)	32.7 (27.2, 38.2)	35.3 (30.2, 40.4)
**Linoleic (g)**	16.3 (12.1, 20.5)	10.1 (4.50, 15.6)	11.4 (6.79, 16.1)	9.57 (6.06, 13.1)
**ALA (g)**	1.74 (0.96, 2.51)	1.43 (0.42, 2.43)	2.14 (1.27, 3.00)	1.14 (0.49, 1.80)
**Very long chain omega-3 fatty acids (mg)**	469 (-403, 1341)	215 (-897, 1326)	1194 (163, 2224)	155 (-581, 892)
**EPA (mg)**	155 (-160, 469)	55.0 (-345, 455)	422 (48.0, 796)	32.0 (-234, 298)

**DPA (mg)**	104 (-49.0, 257)	109 (-87.0, 306)	237 (55.0, 419)	53.0 (-79.0, 185)
**DHA (mg)**	209 (-207, 624)	49.0 (-479, 578)	534 (48.0, 1021)	72.0 (-276, 420)

Data presented as mean (95% CI). Data adjusted for the relevant baseline nutrient intake. **PUFA** polyunsaturated fatty acids; **MUFA** monounsaturated fatty acids; **ALA** α-linolenic acid; **EPA** eicosapentaenoic acid; **DPA** docosapentaenoic acid; **DHA** docosahexaenoic acid. Intervention vs standard care CALD women: * $p < 0.05$.

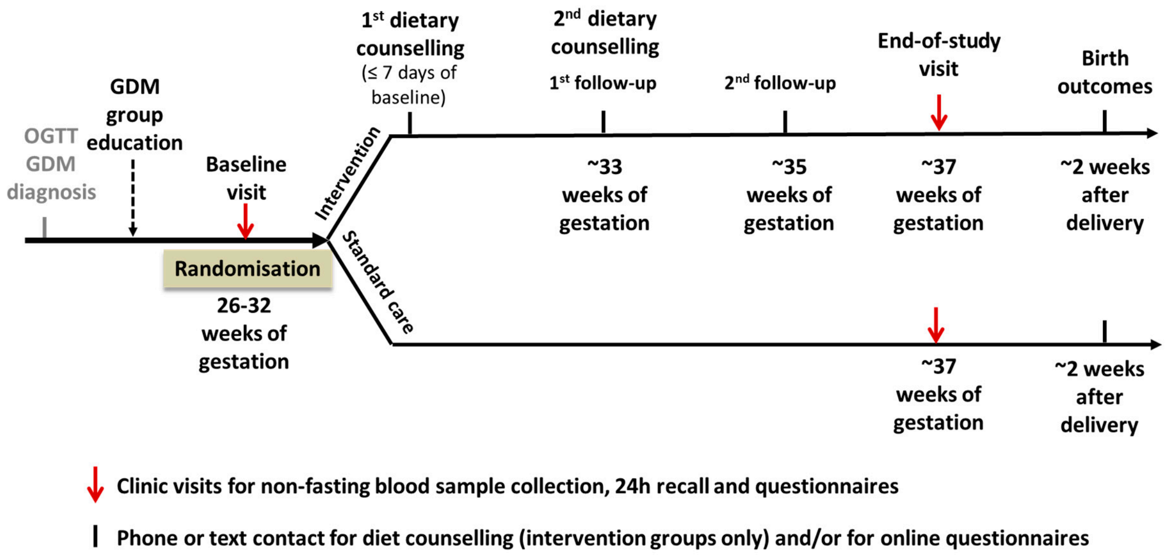

Figure S1. Study timeline

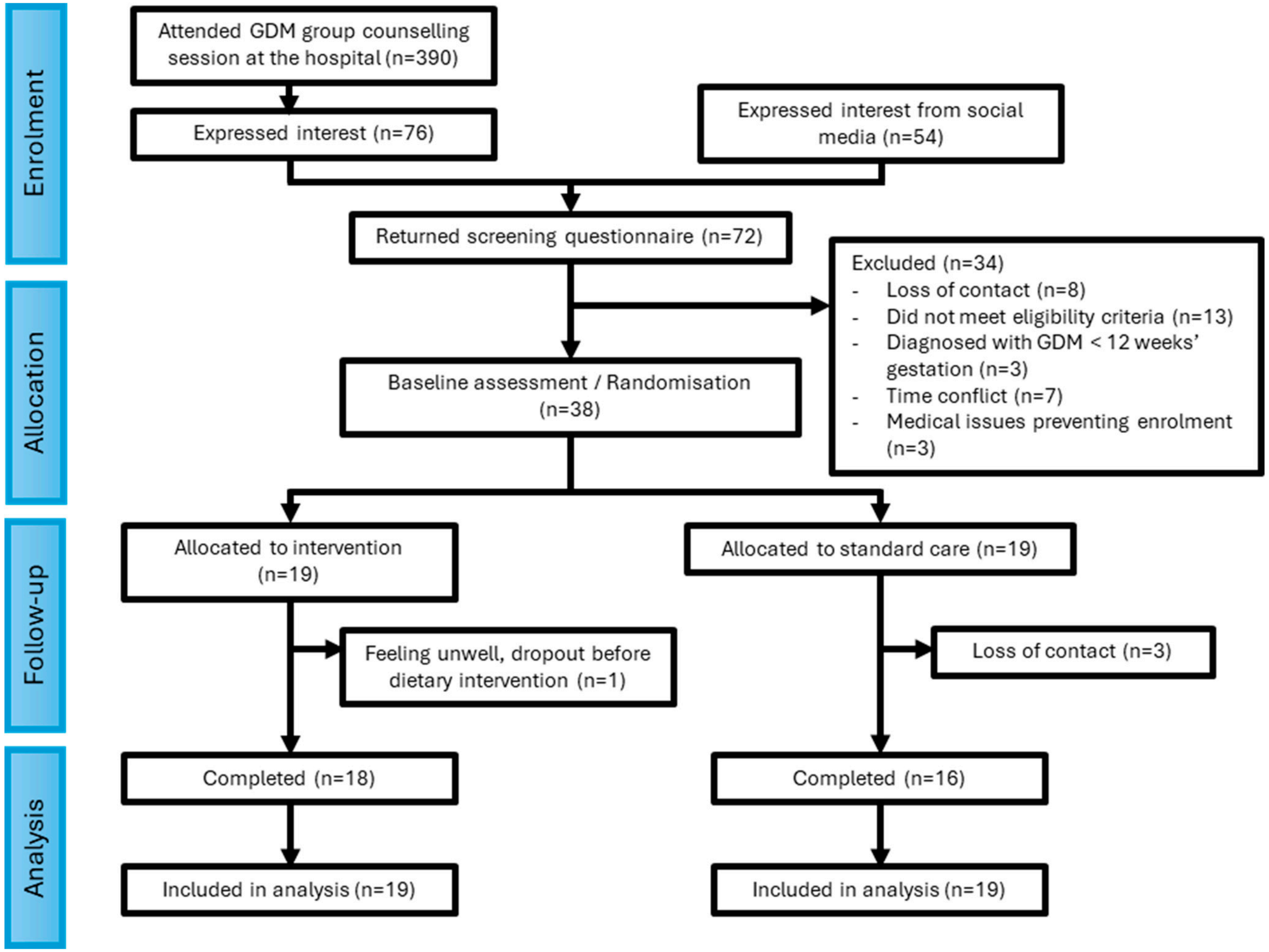

Figure S2. Flow diagram of participants in the study
